# A Prospective Assessment of Helicobacter pylori Eradication Regimens in Treatment-Naïve Patients With Peptic Ulcer Disease

**DOI:** 10.7759/cureus.93665

**Published:** 2025-10-01

**Authors:** Zahra Nawaz, Asma Atta, Samreen Ameen, Shoukat Hussain, Jazba Yousaf, Aiza Ali Akbar, Syeda Wajiha Batool, Abdullah Elrefae, Hifza Ishtiaq, Muhammad Iftikhar Khattak, Tahir Iqbal Mirza

**Affiliations:** 1 Department of ENT, Royal Free Foundation NHS trust, Barnet, GBR; 2 Anaesthesia, Azad Jammu and Kashmir Medical College, Muzaffarabad, PAK; 3 Surgery, Azad Jammu and Kashmir Medical College, Muzaffarabad, PAK; 4 Medicine, Combined Military Hospital, Muzaffarabad, PAK; 5 Department of Geriatric Medicine, Russells Hall Hospital, Dudley, GBR; 6 Department of Trauma and Orthopaedic, AlBashir Hospital, Amman, JOR; 7 Internal Medicine, Abbas Institute of Medical Sciences, Muzaffarabad, PAK; 8 Research and Development, Health Services Academy, Islamabad, PAK; 9 Department of Surgery, Combined Military Hospital, Kharian, PAK

**Keywords:** antibiotic resistance, bismuth quadruple therapy, concomitant therapy, eradication rate, helicobacter pylori, pakistan, peptic ulcer disease, triple therapy

## Abstract

Background: *Helicobacter pylori* (*H. pylori*) infection is a leading cause of peptic ulcer disease (PUD), with eradication complicated by increasing antibiotic resistance.

Objective: To prospectively evaluate and compare the efficacy of various first-line *H. pylori* eradication regimens in treatment-naïve patients diagnosed with PUD.

Methodology: A prospective observational study was conducted from October 2023 to September 2024 at the Department of Gastroenterology, Azad Jammu and Kashmir Medical College and Abbas Institute of Medical Sciences, Muzaffarabad. A total of 252 endoscopically confirmed, treatment-naïve PUD patients were enrolled and assigned to one of the three regimens using a rotational system. Eradication success was evaluated at 6 weeks post-treatment via urea breath or stool antigen test. Antibiotic resistance was assessed using culture and sensitivity testing from gastric biopsies. Logistic regression was used to identify predictors of eradication success.

Results: Among 252 patients, 84 (33.33%) received standard triple therapy, 83 (32.94%) received bismuth-based quadruple therapy, and 85 (33.73%) received concomitant therapy. Overall, *H. pylori* eradication was achieved in 208 patients (82.54%), including 61 (72.62%) in the triple therapy group, 71 (85.54%) in the quadruple group, and 76 (89.41%) in the concomitant group (p=0.01). Resistance to metronidazole (n=131; 52.78%), clarithromycin (n=92; 36.51%), and amoxicillin (n=17; 6.75%) was associated with significantly reduced eradication rates, particularly in dual- and triple-resistant strains. Multivariate analysis confirmed that concomitant therapy (aOR: 2.72, p=0.007) and bismuth-based quadruple therapy (aOR: 2.11, p=0.033) were significantly more effective than standard triple therapy.

Conclusion: Concomitant and bismuth-based quadruple therapies are superior to standard triple therapy in *H. pylori* eradication, particularly in areas with high antibiotic resistance.

## Introduction

Peptic ulcer disease (PUD) remains a prevalent gastrointestinal disorder worldwide, characterized by mucosal erosions in the stomach or duodenum that result in significant morbidity and reduced quality of life [1,2]. One of the most well-established etiological agents of PUD is *Helicobacter pylori* (*H. pylori*), a Gram-negative spiral bacterium that colonizes the gastric mucosa [3]. It has been implicated not only in the pathogenesis of PUD but also in chronic gastritis, mucosa-associated lymphoid tissue (MALT) lymphoma, and gastric carcinoma [4]. Despite the widespread recognition of its clinical relevance, *H. pylori* continues to pose a global health challenge due to evolving antibiotic resistance, regional differences in prevalence, and varying treatment responses [5].

First-line eradication regimens typically combine a proton pump inhibitor (PPI) with two or more antibiotics [6]. Historically, the standard triple therapy consisted of a PPI (such as omeprazole, lansoprazole, or esomeprazole), clarithromycin, and either amoxicillin or metronidazole [7]. Although once highly effective, its eradication rates have declined globally due to rising clarithromycin and metronidazole resistance [8]. To overcome these limitations, several alternative regimens have been introduced. Bismuth-based quadruple therapy includes a PPI, bismuth subsalicylate or subcitrate, tetracycline, and metronidazole, and has shown effectiveness even in regions with high clarithromycin resistance [9]. Concomitant therapy involves a PPI combined with amoxicillin, clarithromycin, and metronidazole given simultaneously, while sequential therapy administers a PPI plus amoxicillin for the first 5 days, followed by a PPI with clarithromycin and metronidazole for the next 5 days [9]. These regimens have demonstrated variable success, largely influenced by local antibiotic resistance patterns, patient adherence, and host factors.

In regions with limited surveillance data on resistance patterns and eradication success, the empirical selection of *H. pylori* eradication therapy becomes particularly problematic [10]. Pakistan, like many developing countries, faces challenges due to high *H. pylori* prevalence and limited accessibility to resistance testing [11]. To guide clinical decision-making, international groups such as the American Gastroenterological Association (AGA) have published expert reviews and practice updates on the management of refractory *H. pylori* infection, emphasizing appropriate regimen selection after treatment failure and highlighting the importance of local resistance patterns in tailoring therapy [12]. Nevertheless, data from low- and middle-income countries remain sparse, underscoring the need for locally relevant evidence. Thus, evaluating the comparative efficacy of available first-line regimens in treatment-naïve patients becomes crucial for evidence-based clinical decision-making.

Understanding real-world treatment outcomes in such populations can help refine empirical therapy recommendations and potentially curb the progression to more severe gastrointestinal pathologies. Additionally, the early and successful eradication of *H. pylori* can reduce the long-term burden on healthcare systems and improve patient outcomes. In Pakistan, despite the high prevalence of *H. pylori* infection, there are currently no comprehensive national guidelines for eradication therapy, and available data remain limited to regional studies. In pediatric patients from Islamabad, 25.9% of cases showed resistance to both clarithromycin and metronidazole [13]. Among adult clinical isolates, resistance rates were remarkably high-73.9% to metronidazole, 47.8% to clarithromycin, and 54.3% to amoxicillin [14]. These data underscore the urgent need for locally relevant evidence to inform empirical treatment strategies.

Research objective

To prospectively evaluate and compare the efficacy of various first-line *H. pylori* eradication regimens in treatment-naïve patients diagnosed with PUD.

## Materials and methods

Study design and setting

This prospective observational study was conducted at the Department of Gastroenterology, Azad Jammu and Kashmir Medical College (AJKMC), and the Abbas Institute of Medical Sciences (AIMS), Muzaffarabad. The study spanned one year, from October 2023 to September 2024. It aimed to evaluate the efficacy of different first-line *H. pylori* eradication regimens in treatment-naïve patients diagnosed with PUD.

Inclusion and exclusion criteria

The study included adult patients aged 18 years and older who presented with endoscopically confirmed gastric or duodenal ulcers and tested positive for *H. pylori* infection through either a urea breath test, stool antigen test, or rapid urease test. Only treatment-naïve individuals - those with no previous history of *H. pylori* eradication - were considered for inclusion. Patients were excluded if they had used antibiotics, PPIs, or bismuth compounds within the last four weeks; had a known history of gastric malignancy or active gastrointestinal bleeding; were pregnant or lactating; had severe systemic illness; or were unable to comply with the treatment or follow-up protocols.

Sample size

A total of 252 patients were enrolled using convenience sampling from the eligible outpatient and inpatient population presenting with PUD at the participating centers. The rationale for using convenience sampling was the multicenter clinical setting and the intent to capture all consecutive treatment-naïve patients meeting the inclusion criteria over the one-year study duration. While a formal a priori sample size calculation was not performed due to the exploratory nature of the study, we subsequently conducted a post hoc power analysis based on the observed eradication rates across treatment groups. This analysis, using an alpha of 0.05, confirmed that our study had >80% statistical power to detect clinically meaningful differences in eradication outcomes, thereby validating the adequacy of the sample size. Notably, our cohort is consistent with or larger than those reported in other prospective observational and interventional studies evaluating *H. pylori* eradication strategies. For example, De Francesco et al. [15] enrolled 131 patients in a randomized trial comparing two new eradication regimens and reported eradication rates of 67.2% and 96.8% with different treatment durations. Similarly, Teima et al. conducted a prospective cohort study on 240 patients, comparing a novel sequential regimen with traditional triple therapy and levofloxacin, omeprazole, nitazoxanide, and doxycycline (LOAD) therapy in both treatment-naïve and previously treated individuals, and demonstrated superior eradication outcomes with sequential therapy [16]. Importantly, in a local context, Asim et al. [17] conducted a randomized controlled trial in Pakistan with 196 participants, showing higher eradication rates with bismuth-based quadruple therapy (93.8%) compared to standard triple therapy (84.6%) on an intention-to-treat basis. Collectively, these findings reinforce that our sample size of 252 patients was not only appropriate but sufficiently powered for an exploratory, real-world evaluation of first-line *H. pylori* eradication regimens.

Data collection

Patients were allocated into four treatment groups using a rotational assignment strategy to minimize physician preference bias and ensure balanced distribution. The regimens were as follows: Standard Triple Therapy (Amoxicillin group): PPI (standard dose, twice daily) + Clarithromycin 500 mg twice daily + Amoxicillin 1 g twice daily for 14 days; Standard Triple Therapy (Metronidazole group): PPI (standard dose, twice daily) + Clarithromycin 500 mg twice daily + Metronidazole 500 mg twice daily for 14 days; Bismuth-Based Quadruple Therapy: PPI (standard dose, twice daily) + Bismuth subsalicylate 525 mg four times daily + Tetracycline 500 mg four times daily + Metronidazole 500 mg three times daily for 14 days; Concomitant Therapy: PPI (standard dose, twice daily) + Clarithromycin 500 mg twice daily + Amoxicillin 1 g twice daily + Metronidazole 500 mg twice daily for 14 days.

At baseline, all patients underwent upper gastrointestinal endoscopy with systematic biopsy sampling. Four biopsy specimens were obtained: two from the antrum and two from the corpus. One set was used for rapid urease testing, while the other set was sent for histopathology and bacterial culture. Culture and sensitivity testing were performed for *H. pylori* isolates, with resistance evaluated against clarithromycin, metronidazole, and amoxicillin. As *H. pylori* is difficult to culture, not all samples yielded successful results; however, cultures were attempted in all cases to maintain protocol consistency. Resistance data were incorporated into subgroup analyses to correlate eradication outcomes with susceptibility patterns.

Eradication success was evaluated six weeks after therapy completion using non-invasive testing (urea breath test or stool antigen test), performed at least two weeks after discontinuation of PPIs to minimize false negatives. A second follow-up at twelve weeks was conducted to assess symptom resolution, recurrence, and ulcer healing. Patients with persistent symptoms or non-healing ulcers on follow-up underwent repeat endoscopy to confirm mucosal healing.

Demographic data (age, sex), ulcer characteristics (gastric vs. duodenal), lifestyle factors (smoking), clinical symptoms, treatment compliance, and adverse effects were recorded using a structured, prevalidated proforma.

Statistical analysis

Data were entered and analyzed using SPSS version 26.0. Descriptive statistics such as means and standard deviations were used for continuous variables, while categorical variables were expressed as frequencies and percentages. Eradication rates between different treatment groups were compared using the chi-square test, and p-values less than 0.05 were considered statistically significant. Logistic regression analysis was performed to identify predictors of treatment success, including regimen type, demographic variables, ulcer location, and smoking status.

Ethical approval

Ethical approval was obtained from the Institutional Review Board of Abbas Institute of Medical Sciences (AIMS), Muzaffarabad (1745/AIMS/2023). Written informed consent was obtained from all participants prior to enrollment. All study procedures were conducted in accordance with the ethical standards of the Declaration of Helsinki. Patient confidentiality was maintained throughout the study by anonymizing data and restricting access to authorized personnel only.

## Results

**Figure 1:**
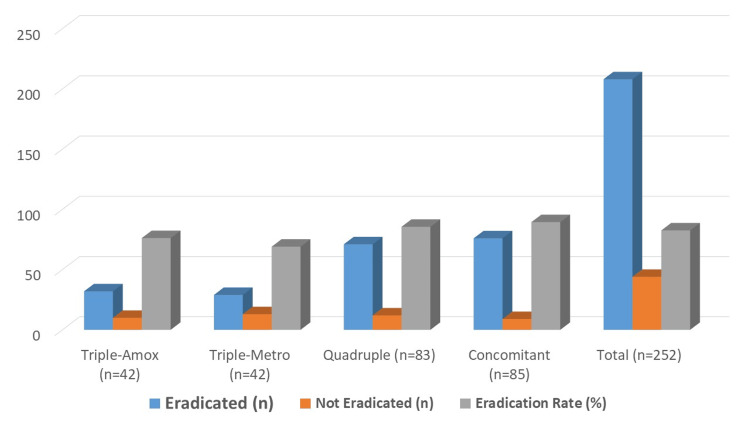
Distribution of patients by treatment regimen

The overall eradication rate in the study cohort was 82.54% (208/252). When stratified by treatment regimen, eradication was highest in the concomitant therapy group (89.41%, 76/85), followed by the bismuth-based quadruple group (85.54%, 71/83), the amoxicillin-based triple group (76.19%, 32/42), and the metronidazole-based triple group (69.05%, 29/42). The 95% confidence intervals (95% CIs) for these estimates overlapped, indicating that the observed differences in eradication rates between regimens were not statistically significant (Figure 1).

Baseline demographic and clinical characteristics were comparable across all four groups (Table 1). The mean age ranged from 41.5 to 43.3 years, with no significant differences by sex distribution. Duodenal ulcers predominated (59.5-68.2%), particularly within the bulb, followed by gastric ulcers, most commonly in the antrum. Ulcer size was <1 cm in approximately two-thirds of cases across regimens. Smoking prevalence was between 22.4% and 28.6%. The most frequent presenting symptoms were epigastric pain (91.6-95.2%) and bloating (69.1-74.1%). No statistically significant intergroup differences were observed, confirming group comparability at baseline.

**Table 1:** Baseline Demographic and Clinical Characteristics by Treatment Group

Category	Characteristic	Triple-Amox (n=42)	Triple-Metro (n=42)	Quadruple (n=83)	Concomitant (n=85)	p-value
Demographics	Mean Age (years)	42.3 ± 11.2	41.9 ± 11.6	43.3 ± 10.9	41.5 ± 12.1	0.61
	Male (n; %)	23 (54.76%)	24 (57.14%)	44 (53.01%)	42 (49.41%)	0.92
	Female (n; %)	19 (45.24%)	18 (42.86%)	39 (46.99%)	43 (50.59%)	0.92
Ulcer Type	Duodenal Ulcer (n; %)	27 (64.29%)	25 (59.52%)	54 (65.06%)	58 (68.24%)	0.69
	Gastric Ulcer (n; %)	15 (35.71%)	17 (40.48%)	29 (34.94%)	27 (31.76%)	0.69
Ulcer Location	Duodenal bulb (%)	22 (52.38%)	21 (50.00%)	45 (54.22%)	47 (55.29%)	0.83
	Duodenal superior wall (%)	5 (11.90%)	4 (9.52%)	9 (10.84%)	11 (12.94%)	0.91
	Gastric antrum (%)	9 (21.43%)	11 (26.19%)	17 (20.48%)	15 (17.65%)	0.88
	Gastric body (%)	6 (14.29%)	6 (14.29%)	12 (14.46%)	12 (14.12%)	0.99
Ulcer Size	< 1 cm (%)	28 (66.67%)	27 (64.29%)	55 (66.27%)	59 (69.41%)	0.91
	≥ 1 cm (%)	14 (33.33%)	15 (35.71%)	28 (33.73%)	26 (30.59%)	0.91
Lifestyle	Smokers (n; %)	11 (26.19%)	12 (28.57%)	20 (24.10%)	19 (22.35%)	0.74
Clinical Symptoms	Bloating Present (n; %)	29 (69.05%)	30 (71.43%)	61 (73.49%)	63 (74.12%)	0.85
	Epigastric Pain (n; %)	40 (95.24%)	39 (92.86%)	76 (91.57%)	78 (91.76%)	0.76

Overall eradication was achieved in 208 of 252 patients (82.5%), with significant variation between regimens (Table 2). The highest eradication rate was observed in the concomitant group (89.4%), followed by quadruple therapy (85.5%). Triple-amoxicillin achieved 76.2%, while triple-metronidazole had the lowest rate at 69.1%. The difference in eradication success across groups reached statistical significance (p=0.03). The 95% confidence intervals (95% CIs) for each group overlapped but still reflected a clear trend favoring concomitant and quadruple regimens over triple therapy.

**Table 2:** Helicobacter pylori Eradication Rates at 6 Weeks by Regimen

Treatment Regimen	Eradicated (n)	Not Eradicated (n)	Eradication Rate (%)	p-value
Triple-Amox (n=42)	32	10	76.19	0.03
Triple-Metro (n=42)	29	13	69.05	
Quadruple (n=83)	71	12	85.54	
Concomitant (n=85)	76	9	89.41	
Total (n=252)	208	44	82.54	

Clinical outcomes at 12 weeks are summarized in Table 3. Symptom resolution was highest with concomitant therapy (90.6%) and quadruple therapy (85.5%), compared with 78.6% and 81.0% in the amoxicillin- and metronidazole-based triple therapy groups, respectively. Symptom recurrence was least frequent in the concomitant group (9.4%). Repeat endoscopy, performed in about one-third of patients, confirmed ulcer healing in over 90% of concomitant and quadruple therapy recipients, compared with 80-86% in triple therapy groups. Although these outcomes favored the more intensive regimens, differences did not reach statistical significance (p>0.05), and 95% CIs for healing outcomes overlapped between groups.

**Table 3:** Clinical Outcomes at 12-Week Follow-Up

Outcome	Triple-Amox (n=42)	Triple-Metro (n=42)	Quadruple (n=83)	Concomitant (n=85)	p-value
Symptom Resolution (n; %)	33 (78.57%)	34 (80.95%)	71 (85.54%)	77 (90.59%)	0.08
Symptom Recurrence (n; %)	9 (21.43%)	8 (19.05%)	12 (14.46%)	8 (9.41%)	0.06
Repeat Endoscopy Done (%)	15 (35.71%)	14 (33.33%)	31 (37.35%)	28 (32.94%)	0.84
Ulcer Healing Observed (%)	12 (80.00%)	12 (85.71%)	29 (93.55%)	27 (96.43%)	0.11

Treatment adherence was high across all regimens (Table 4). Good compliance ranged from 85.7% in the triple-amoxicillin group to 92.9% in the concomitant group. Mild side effects were the most common adverse events (21.4-28.2%), while moderate to severe side effects were infrequent (3.5-7.1%). Treatment discontinuation occurred in ≤4.8% of patients across groups. None of these differences were statistically significant (p>0.05), indicating overall tolerability and feasibility of all regimens in clinical practice.

**Table 4:** Treatment Compliance and Adverse Effects

Category	Parameter	Triple-Amox (n=42)	Triple-Metro (n=42)	Quadruple (n=83)	Concomitant (n=85)	p-value
Compliance	Good Compliance (n; %)	36 (85.71%)	37 (88.10%)	76 (91.57%)	79 (92.94%)	0.36
Adverse Effects	Mild Side Effects (n; %)	9 (21.43%)	10 (23.81%)	22 (26.51%)	24 (28.24%)	0.55
	Moderate-Severe (%)	2 (4.76%)	3 (7.14%)	4 (4.82%)	3 (3.53%)	0.68
Treatment Completion	Discontinuation (n; %)	1 (2.38%)	2 (4.76%)	2 (2.41%)	2 (2.35%)	0.91

**Figure 2:**
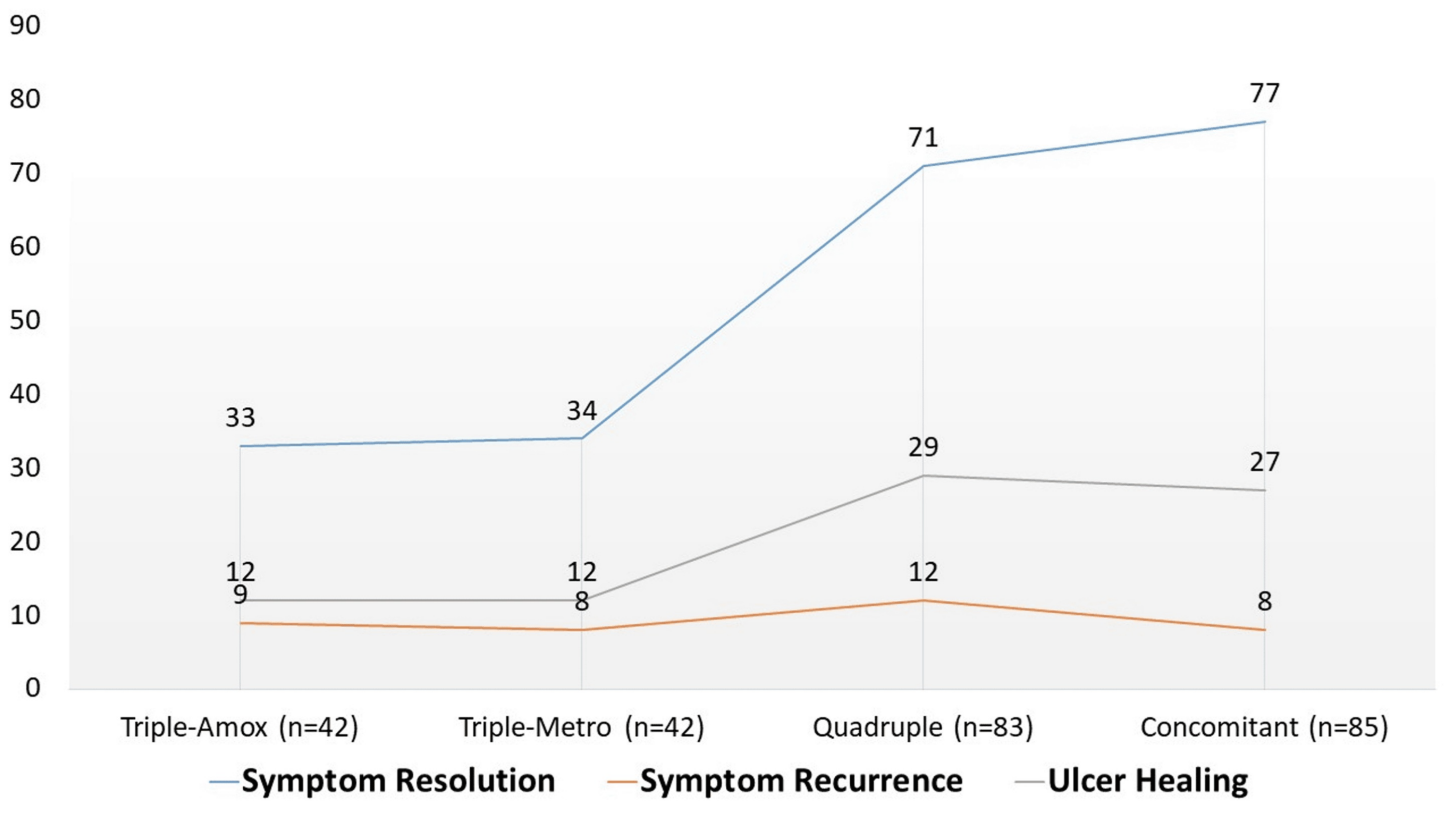
Grouped bar chart of clinical outcomes (symptom resolution, recurrence, ulcer healing) at 12 weeks.

At 12-week follow-up, symptom resolution was most frequently observed in the concomitant therapy group (90.59%, 77/85), followed by quadruple therapy (85.54%, 71/83), triple-metronidazole (80.95%, 34/42), and triple-amoxicillin (78.57%, 33/42), as shown below in Figure 2. Symptom recurrence was lowest in the concomitant group (9.41%) compared to 14.46% in the quadruple, 19.05% in the triple-metronidazole, and 21.43% in the triple-amoxicillin groups. Endoscopic evaluation in a subset of patients revealed higher ulcer healing rates in quadruple (93.55%) and concomitant regimens (96.43%) compared to triple therapy arms (80.00% and 85.71%). While these differences favored quadruple and concomitant regimens, statistical significance was not achieved (p>0.05).

Eradication outcomes varied significantly across resistance patterns and treatment regimens. In clarithromycin-sensitive strains, all regimens achieved high success rates (>83%), with quadruple and concomitant therapies performing slightly better. However, in clarithromycin-resistant strains, eradication fell sharply in both triple therapy arms (50.0% and 33.3%), while concomitant therapy maintained the highest success (87.5%) (Table 5). Similar trends were observed for metronidazole and amoxicillin resistance, where quadruple and concomitant therapies preserved moderate-to-high efficacy, in contrast to markedly lower rates with triple regimens. Dual and triple resistance were associated with the poorest outcomes overall, yet concomitant therapy still showed comparatively higher eradication rates.

**Table 5:** Helicobacter pylori Eradication Rates Stratified by Antibiotic Resistance Patterns and Treatment Regimen Values are n/N (%). Resistance testing was available for 216 patients. P-values indicate overall significance across groups. Eradication success is strongly reduced in resistant strains.

Resistance Pattern	Triple-Amox (n=42)	Triple-Metro (n=42)	Quadruple (n=83)	Concomitant (n=85)	p-value
Clarithromycin-Sensitive	34/38 (89.5%)	30/36 (83.3%)	52/57 (91.2%)	62/69 (89.9%)	<0.001
Clarithromycin-Resistant	2/4 (50.0%)	2/6 (33.3%)	15/26 (57.7%)	14/16 (87.5%)	
Metronidazole-Sensitive	35/38 (92.1%)	28/30 (93.3%)	54/61 (88.5%)	60/66 (90.9%)	0.033
Metronidazole-Resistant	3/4 (75.0%)	4/12 (33.3%)	13/22 (59.1%)	16/19 (84.2%)	
Amoxicillin-Sensitive	33/36 (91.7%)	32/37 (86.5%)	56/67 (83.6%)	60/71 (84.5%)	0.023
Amoxicillin-Resistant	3/6 (50.0%)	2/5 (40.0%)	12/16 (75.0%)	18/21 (85.7%)	
Dual Resistance	2/5 (40.0%)	1/6 (16.7%)	8/14 (57.1%)	13/18 (72.2%)	<0.001
Triple Resistance	0/2 (0.0%)	0/3 (0.0%)	1/5 (20.0%)	3/7 (42.9%)	

Multivariate logistic regression confirmed treatment regimen and antibiotic resistance as the strongest predictors of eradication success. Compared with triple-amoxicillin therapy, quadruple (aOR 2.11, 95% CI 1.06-4.18, p=0.033) and concomitant regimens (aOR 2.72, 95% CI 1.31-5.65, p=0.007) were independently associated with significantly higher eradication rates, while triple-metronidazole therapy did not differ significantly (Table 6). Clarithromycin, metronidazole, and amoxicillin resistance were each strong negative predictors of treatment success, with adjusted odds ratios ranging from 0.28 to 0.42. In contrast, age, sex, and smoking status were not significantly associated with eradication outcomes.

**Table 6:** Multivariate Logistic Regression Analysis for Predictors of Helicobacter pylori Eradication Success aOR: adjusted odds ratio; CI: confidence interval. Model adjusted for age, sex, smoking, ulcer site, and regimen type. Quadruple and concomitant therapies were significantly associated with higher eradication rates, while antibiotic resistance strongly predicted failure

Predictor Variable		aOR (95% CI)	p-value
Treatment Regimen	Triple-Amox (reference)	1.00	–
	Triple-Metro	0.81 (0.35–1.86)	0.62
	Quadruple	2.11 (1.06–4.18)	0.033
	Concomitant	2.72 (1.31–5.65)	0.007
Clarithromycin Resistance		0.38 (0.21–0.69)	0.001
Metronidazole Resistance		0.42 (0.19–0.91)	0.029
Amoxicillin Resistance		0.28 (0.09–0.82)	0.021
Age ≥50 Years		0.93 (0.51–1.70)	0.81
Male sex		1.08 (0.59–1.97)	0.79
Smoking		0.84 (0.45–1.58)	0.59

## Discussion

This prospective observational study evaluated the comparative efficacy of three first-line *H. pylori* eradication regimens - standard triple therapy, bismuth-based quadruple therapy, and concomitant therapy - in treatment-naïve patients with PUD. The overall eradication rate across all regimens was 82.54%, which is consistent with global trends but underscores ongoing variability in treatment outcomes due to antibiotic resistance and regional prescribing practices.

Concomitant therapy achieved the highest eradication rate at 89.41%, followed by bismuth quadruple therapy at 85.54% and standard triple therapy at 72.62% (p=0.01). These results mirror those of a previous study, which reported eradication rates of 89.41% for concomitant therapy, 85.54% for bismuth-based quadruple therapy, and 72.62% for standard triple therapy in treatment-naïve PUD patients [18]. Similarly, a Pakistani study by Ahmed et al. observed a 72.62% eradication rate for standard triple therapy, comparable to our findings, thereby reinforcing local data and highlighting the declining effectiveness of this regimen in regions with high clarithromycin resistance [19]. The incorporation of antibiotic sensitivity profiling in our study further strengthens the evidence that resistance is a critical determinant of treatment success.

In our cohort, resistance was most prevalent for metronidazole (52.78%), followed by clarithromycin (36.51%) and amoxicillin (6.75%). These rates are in line with earlier surveillance reports, where clarithromycin resistance frequently exceeds 30% and is a major contributor to triple therapy failure [20]. Patients with clarithromycin-sensitive strains had significantly higher eradication rates (90.00%) compared to resistant cases (69.57%, p<0.001). Comparable differences were observed for metronidazole (91.60% vs. 74.44%) and amoxicillin (84.26% vs. 58.82%). Dual resistance to clarithromycin and metronidazole reduced eradication success to 66.18%, while triple resistance lowered the rate to 33.33%, corroborating prior studies reporting poor outcomes in multi-drug-resistant strains [21].

Logistic regression analysis confirmed that concomitant therapy (aOR: 2.72; p=0.007) and bismuth quadruple therapy (aOR: 2.11; p=0.033) were significantly more effective than triple therapy, even after adjustment for demographic and clinical variables. These results are consistent with previous research advising against empirical triple therapy in areas where clarithromycin resistance exceeds 15% [22]. Notably, factors such as age, gender, smoking status, and ulcer location did not significantly influence eradication outcomes, echoing findings from earlier studies and suggesting that bacterial and pharmacological factors outweigh host demographics in determining treatment success [23].

Symptom resolution and ulcer healing rates were highest in the concomitant therapy group (90.59% and 96.43%, respectively), underscoring the clinical impact of microbiological eradication. Collectively, these findings support the broader adoption of regimens with higher efficacy and tolerability in empiric treatment protocols.

Study strengths and limitations

A major strength of this study is its prospective multicenter design, which improves generalizability by reflecting real-world clinical practice across two tertiary care centers. The inclusion of antibiotic susceptibility testing allowed us to directly correlate resistance patterns with eradication outcomes, an approach that is often lacking in similar studies. Furthermore, the use of rotational assignment of regimens minimized physician preference bias, while baseline endoscopic and histological confirmation ensured diagnostic accuracy. The sample size of 252 patients, validated through post hoc power analysis (>80% at α = 0.05), was sufficient to detect clinically meaningful differences in treatment efficacy.

However, certain limitations must be acknowledged. First, the non-randomized design may introduce residual confounding despite balanced allocation. Second, while the sample size was adequate for overall comparisons, it may not have been sufficient for detailed subgroup analyses, particularly among patients with rare resistance patterns (e.g., triple resistance). Third, although baseline endoscopy was performed in all patients, repeat endoscopy was conducted in only 34.9% of participants-those with persistent symptoms or non-healing ulcers-potentially limiting ulcer healing data. Lastly, eradication success was primarily assessed with non-invasive tests (urea breath or stool antigen), which, while validated, may slightly under- or overestimate eradication compared with universal repeat endoscopy.

## Conclusions

This study demonstrates that concomitant and bismuth-based quadruple therapies are significantly more effective than standard triple therapy for *H. pylori* eradication, particularly in regions with high clarithromycin and metronidazole resistance. Antibiotic resistance patterns were strong predictors of treatment failure, emphasizing the importance of local resistance surveillance in guiding empiric therapy. Our findings support replacing standard triple therapy with more robust regimens in areas with rising resistance, aligning with current international guidelines. Tailored treatment strategies, including susceptibility-based regimens, may further enhance eradication rates and reduce the burden of PUD in resource-limited settings.
